# Pathways of IL-1β secretion by macrophages infected with clinical *Mycobacterium tuberculosis* strains^☆^

**DOI:** 10.1016/j.tube.2013.05.002

**Published:** 2013-09

**Authors:** Nitya Krishnan, Brian D. Robertson, Guy Thwaites

**Affiliations:** a MRC Centre for Molecular Bacteriology and Infection, Department of Medicine, Imperial College London, South Kensington Campus, London SW7 2AZ, UK; b Centre for Clinical Infection and Diagnostics Research, Department of Infectious Diseases, King's College London, Guy's and St. Thomas' Hospitals NHS Foundation Trust, London SE1 9RT, UK

**Keywords:** Mycobacteria, Interleukin-1β, Macrophages, Serine proteases, Caspase-1

## Abstract

The pro-inflammatory cytokine IL-1β is a key mediator of inflammation and plays an important role in the host resistance to *Mycobacterium tuberculosis* infections. To date, most studies have examined the mechanisms of IL-1β secretion using laboratory strains of *M. tuberculosis* and the findings may not be widely applicable to contemporary clinical strains. Here, we investigated the primary pathways of IL-1β secretion in macrophages infected with a panel of 17 clinical *M. tuberculosis* isolates, representing Euro-American, Indo-Oceanic and East-Asian/Beijing lineages. Our aim was to dissect the pathways involved in *M. tuberculosis* induced IL-1β secretion and to determine whether they are common to all clinical isolates. We found that the isolates were capable of eliciting variable concentrations of IL-1β from infected murine macrophages, but this phenomenon could not be attributed to differential IL-1β mRNA transcription or pro-IL-1β accumulation. We demonstrate that viable bacteria are required to induce IL-1β secretion from macrophages, but IL-1β secretion was only partially abrogated by caspase-1 inhibition. Almost complete IL-1β secretion inhibition was produced with combined caspase-1 and some serine protease inhibitors. Taken together, these findings demonstrate that clinical strains of *M. tuberculosis* employ a unique caspase-1 independent pathway to stimulate IL-1β secretion from macrophages.

## 1 Introduction

*Mycobacterium tuberculosis* is a complex pathogen capable of subverting the immune response and establishing life-long persistent infection in the host.^1^ Detailed understanding of the host–pathogen interaction still remains to be delineated. Macrophages, the primary host cells for mycobacteria, respond to *M. tuberculosis* infection by up regulating a variety of cytokines including TNF-α, IL-6 and IL-1β. Murine models of tuberculosis provide compelling evidence for the importance of IL-1β signalling in the host resistance to *M. tuberculosis* infection. IL-1 receptor (IL-1R) and IL-1β knock-out mice are more susceptible to *M. tuberculosis* infection, exhibiting high bacterial burden in the lungs and increased mortality early in infection.^2–5^

**Figure 1:**
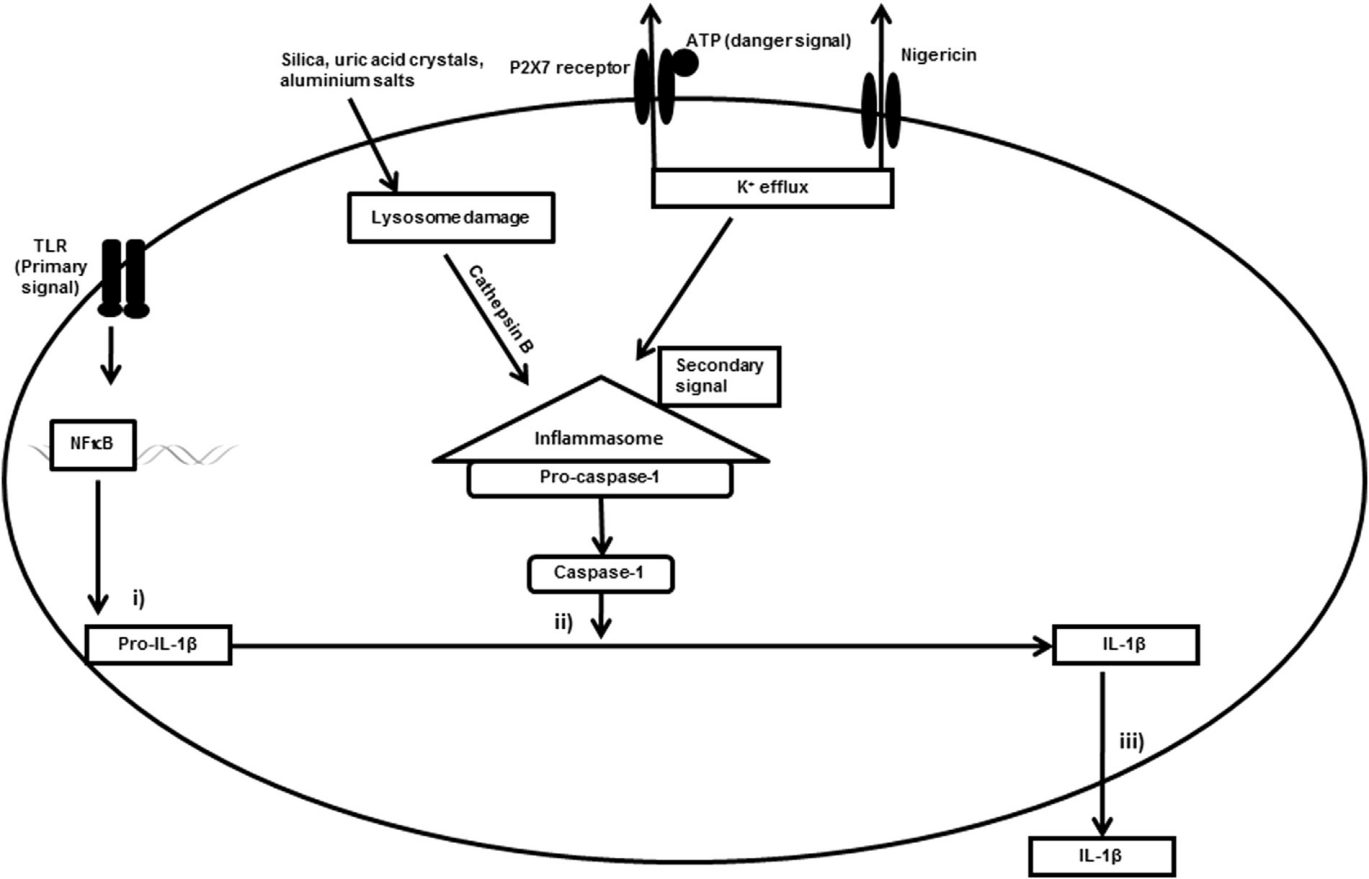
Diagram illustrating the different signalling pathways of IL-1β induction in macrophages. The transcription, translation and processing of IL-1β is a highly regulated process comprising of three major steps: i) synthesis of pro-IL-1β regulated by pattern recognition receptors like TLRs, ii) cleavage of pro-IL-1β by caspase-1 resulting in the mature form of IL-1β and iii) release of mature IL-1β into the external environment. Activation of the inflammasome by secondary signals in the form of microbial ligands, Nigericin, ATP and other danger signals (e.g. silica, uric acid crystals) induces activation of caspase-1.

The processes involved in the transcription, processing and release of IL-1β from macrophages are tightly controlled (summarised in Figure 1). Control of gene expression is mediated by a number of processes including negative regulation of IL-1β by type I interferons, which are induced by *M. tuberculosis*.^6,7^ The engagement of pathogen associated molecular patterns (PAMPs) with pattern recognition receptors (PRRs) such as toll-like receptors (TLRs) acts as a primary signal leading to the synthesis of the inactive 31-kDa pro-IL-1β in macrophages.^8^ Primary signalling via TLRs is insufficient to stimulate the release of active IL-1β. Additional microbial danger signals are required for the processing and release of the mature form of IL-1β. Cleavage of pro-IL-1β into the biologically active 17 kDa IL-1β protein requires the activation of the inflammasome, a multi-protein complex which stimulates caspase-1 activation to promote the processing and secretion of pro-inflammatory cytokines. The inflammasome provides a platform for the conversion of caspase-1 precursor into its active form in response to signalling via the nucleotide oligomerization domain (NOD) receptors and danger associated molecular patterns such as ATP.^9,10^ Activated caspase-1 cleaves pro-IL-1β into the mature biologically active 17 kDa form that is then rapidly secreted out of the cell via an incompletely understood mechanism. A recent study has elegantly shown that nitric oxide produced by IFN-γ stimulated macrophages regulates the processing of pro-IL-1β by directly acting on the inflammasome.^11^ In other cell types like dendritic cells, mycobacteria are reported to activate the caspase-8 dependent inflammasome.^12^ The control of processing and secretion of IL-1β seems to be complex process dependent on multiple sensors and effectors.

Several studies have stressed the importance of the mycobacterial region of difference-1 (RD-1) locus in modulating the secretion of IL-1β by infected cells. *M. marinum* ΔRD-1 and *M. bovis* BCG lack functional ESX-1 secretion systems and are impaired in their ability to induce secretion of mature IL-1β *in vitro.*^13–15^ A functional ESX-1 secretion system has been implicated in the transfer of bacterial products like the early secreted antigen-6 (ESAT-6), a known stimulator of caspase-1.^16^ Effector proteins secreted via the ESX-1 secretion system are thought to be involved in triggering a potassium ion efflux which is necessary for the activation of caspase-1 during *M. tuberculosis* infection.^14^

The contribution of cell death to the regulation of immune pathways during *M. tuberculosis* infection is not clear. Studies have reported the occurrence of both apoptotic and necrotic cell death during *M. tuberculosis* cellular infection. Apoptotic cell death may limit the spread of intracellular bacteria^17,18^; while, necrotic cell death may aid in the escape and dissemination of the bacteria into new host cells. Induction of necrotic cell death closely correlates with bacterial burden and virulence of the infecting *M. tuberculosis* strain,^19,20^ with evidence that necrosis may be mediated by ESX-1 activation of the inflammasome – NOD-like receptor family, pryin domain containing 3 (NLRP3).^21^ Under conditions of extreme inflammatory stress, increased levels of cell death contributes to plasma membrane damage which can lead to the rapid release of bioactive IL-1β from immune cells.^22^

Increasingly, genetic diversity in *M. tuberculosis* is understood to influence aspects of the host–pathogen interaction, including virulence, immune modulation and clinical outcomes.^23,24^ Most studies to date have examined the mechanisms of IL-1β secretion using laboratory strains of *M. tuberculosis* like H37Rv.^5,6,25^ Here, we investigated the primary pathways of IL-1β secretion in macrophages infected with a panel of clinical isolates of *M. tuberculosis*. In this study, we report that the ability of *M. tuberculosis* strains to induce secretion of IL-1β in murine macrophages is dependent on both caspase-1 and serine proteases. Additionally, we report that IL-1β secretion during *M. tuberculosis* infection is independent of cell death in infected macrophages.

## 2 Materials and methods

### 2.1 Bacterial strains and culture conditions

*M. tuberculosis* strains were selected using the criteria previously outlined.^23^ We selected isolates representative of the three major lineages including seven East Asian/Beijing strains (strains 119, 345, 212, 649, 374, 333, 411), four Indo-Oceanic strains (strains 346, 232, 372, 281) and six Euro-American strains (strains 293, 173, 355, 318, 440, 639).

Strains were cultured in Middlebrook 7H9 liquid medium (Becton Dickinson, United Kingdom) supplemented with 0.2% glycerol, 0.05% Tween 80 and 10% oleic acid-albumin-dextrose-catalase (OADC; Becton Dickinson, United Kingdom). For growth on solid medium, Middlebrook 7H10 plates were supplemented with 0.5% glycerol and 10% OADC. Heat-killed strains were prepared by boiling mid-log phase cultures for 20 min before removing from the BSL3 lab.

### 2.2 Isolation of mouse bone marrow macrophages

Bone marrow cells from 8 to10 week old female BALB/c mice were isolated and differentiated into macrophages for 7 days in RPMI 1640 (Lonza, United Kingdom) supplemented with 1 mM sodium pyruvate (Lonza, United Kingdom), 2 mM l-glutamine (Lonza, United Kingdom), 0.05 M, 2-mercaptoethanol (Invitrogen, United Kingdom), 10% heat-inactivated fetal bovine serum (Biosera, United Kingdom) and 20% L-cell conditioned medium (a kind gift from Anne O'Garra, MRC National Institute for Medical Research, London). On day 4, cells were fed with an additional 10 ml of medium. After 7 days in culture, cells were washed with phosphate-buffered saline (PBS) and seeded into 24-well plates at 5 × 10^5^ cells/well or in 48-well plates at 2 × 10^5^ cells/well.

### 2.3 Cellular infection assays

Macrophages were infected with *M. tuberculosis* from mid-log phase cultures at a multiplicity of infection (MOI) of 5. Supernatants were removed at 24 and 48 h post-infection and filtered using 0.22 μm filters (Millipore, United Kingdom) before removing from the BSL3 lab. Concentration of cytokines in the supernatants was determined using an enzyme-linked immunosorbent assay (ELISA) according to the manufacturer's instructions (E-bioscience, United Kingdom). Cell death was assayed using the cell death detection ELISA (Roche Diagnostics, United Kingdom). Briefly, macrophages were lysed following infection and apoptotic cell death was evaluated in the cytoplasmic fractions according to the manufacturer's instructions. Intracellular growth of *M. tuberculosis* was also determined in the lysed cells, once extracellular bacteria had been killed by the addition of streptomycin, for strains 411, 212, 333, 281, 232, 372, 355, 293, 318 over 7 days of infection.

For studies using inhibitors, macrophages were stimulated with the caspase-1 inhibitor, Ac-Tyr-Val-Ala-Asp-2,6-dimethylbenzoyloxymethyl ketone (Ac-YVAD-AOM; Merck Chemicals, United Kingdom) at a concentration of 20 μM, serine protease inhibitor, N-tosyl-l-phenylalanine chloromethylketone (TPCK; Merck Chemicals, United Kingdom) at a concentration of 50 μM, N-tosyl-Lys-Chloromethyl Ketone Hydrochloride (TLCK; Merck Chemicals, United Kingdom) at a concentration of 50 μM, BAY 11-7082 (NFκB inhibitor) at a concentration of 10 μM, and Nigericin (Sigma–Aldrich, United Kingdom) at a concentration of 5 μM. Each *M. tuberculosis* strain was assayed in triplicate in at least two independent experiments.

### 2.4 Real-time PCR

Macrophages were lysed using TRIzol (Invitrogen, United Kingdom) and total RNA was isolated using Purelink RNA mini kit (Invitrogen, United Kingdom), according to the manufacturer's protocol. cDNA was generated from 1 μg total RNA using superscript III reverse transcriptase and random hexamers (Invitrogen, United Kingdom) according to the manufacturer's instructions. Reaction mixtures without reverse transcriptase were used as the negative control. The reaction mix was incubated at 25 °C for 10 min, 50 °C for 30 min, 85 °C for 5 min, followed by an incubation at 37 °C for 20 min, after the addition of RNase H to terminate the reaction. For the real-time PCR, each 10 μl reaction mix contained 5 μl TaqMan universal PCR master mix (Applied Biosystems, United Kingdom), 0.5 μl TaqMan primers and probe, 1 μl cDNA and 3.5 μl of water (Invitrogen, United Kingdom), as recommended by the manufacturer. The reactions were performed on an ABI 7500 real-time PCR thermal cycler (Applied Biosystems, United Kingdom) and the samples were incubated at 95 °C for 20 s, followed by 40 cycles of 95 °C for 3 s and 60 °C for 30 s. Commercially available primers for 18s and IL-1β were purchased from Applied Biosystems and used as recommended by the manufacturer. Each sample was assayed in duplicates on two biological replicates. Parallel reactions with no template controls and reverse transcriptase negative samples were included in each run. Standard curves were generated to calculate the relative copy numbers of each gene. For each gene, the calculated threshold cycle (Ct) of each cytokine transcript was divided by the Ct of the endogenous control, 18s to obtain a normalised number for the cytokine transcript for each strain. Fold change for each gene was calculated relative to the uninfected control. Under the conditions assayed the levels of 18s was constant and thus was chosen as the endogenous control.

### 2.5 Western blots

Macrophages were lysed using RIPA buffer (Sigma–Aldrich, United Kingdom) supplemented with complete protease inhibitor tablets (Roche Diagnostics, United Kingdom). The samples were boiled and separated on a 12% polyacrylamide gel (Invitrogen, United Kingdom) and transferred onto a nitrocellulose membrane (GE healthcare, United Kingdom). The membrane was probed with goat polyclonal antibody for pro-IL-1β, mouse monoclonal antibody for pro-caspase-1 and mouse monoclonal antibody for β-actin. All antibodies were sourced from Santa Cruz Biotechnology, CA, USA. Stripping of the membrane was carried out using restore plus western blot stripping buffer (Thermo Fisher Scientific, United Kingdom) for subsequent probing with β-actin. Concentration of total protein was determined using the BCA protein assay kit (Thermo Fisher Scientific, United Kingdom).

### 2.6 Statistical analysis

All data are presented as mean ± SD of two or three independent experiments. Data was combined and analysed using Mann Whitney *U* test and a *p*-value <0.05 was considered statistically significant. Correlation between IL-1β and cell death was performed using the Spearman rank for correlation. All the statistical tests were performed using Graphpad Prism.

## 3 Results

### 3.1 *M. tuberculosis* clinical isolates induce differential IL-1β secretion from macrophages

**Figure 2:**
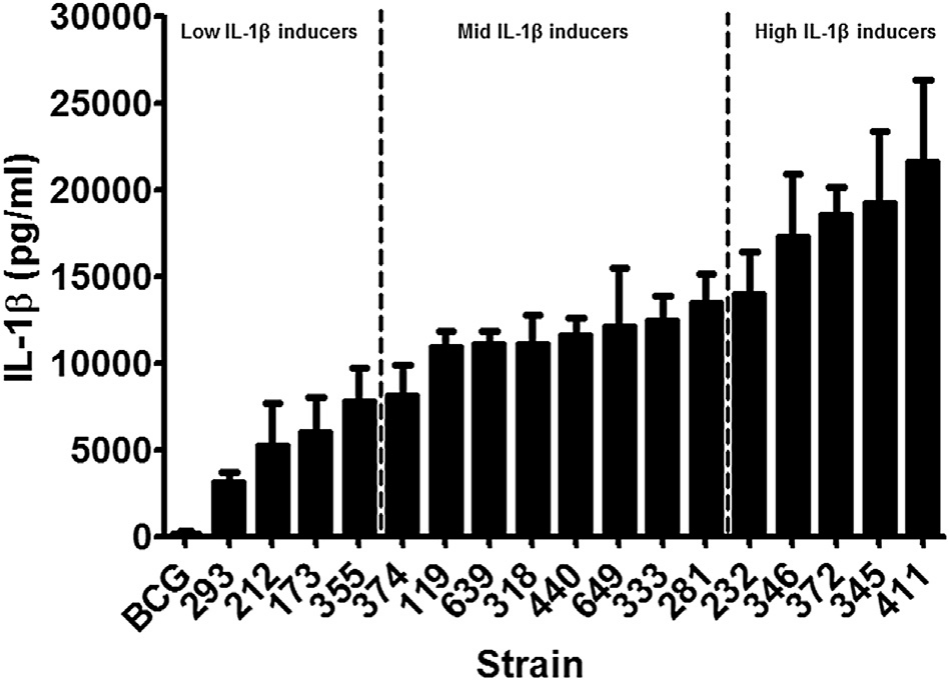
*M. tuberculosis* genotype influences secretion of IL-1β from macrophages. Bone marrow macrophages were infected with 17 clinical isolates of *M. tuberculosis* at a MOI of 5 for 72 h. Cytokine concentrations in the culture supernatants were determined by ELISA. Data shown are the means ± SD of three independent experiments.

We first determined whether *M. tuberculosis* clinical isolates vary in their ability to induce IL-1β secretion from macrophages. Macrophages were infected at a MOI of 5 over 72 h and the concentration of IL-1β was determined by ELISA. Strains of *M. tuberculosis* differed in their ability to promote secretion of IL-1β, with a five-fold difference between the highest and lowest IL-1β inducers (Figure 2). These differences did not correlate with bacterial growth rate within macrophages. Intracellular bacterial growth was measured in 9 of the strains representative of the extremes of IL-1β expression, and in two independent experiments. The growth rates varied between the strains over the first 48 h from 2.0 to 8.7 × 10^3^ CFU/h, but there was no correlation between growth rate and IL-1β expression (correlation coefficient −0.15, *p* = 0.71).

In comparison to clinical isolates of *M. tuberculosis*, BCG was less effective at inducing IL-1β, producing negligible amounts of the cytokine from macrophages. In addition, we also assessed the role of bacterial burden in the secretion of IL-1β in strains 293, 212, 318, 232, 372 and 411. A higher bacillary load (MOI 10) of *M. tuberculosis* was effective in stimulating higher concentrations of IL-1β in comparison to a lower bacterial burden (MOI 1) (data not shown). The fold increased secretion of IL-1β at a MOI 10 compared to MOI 1 was in the range 1.4–2.2 with a median of 1.8 at 24 h and a fold increase in the range of 1.3–2.4 with a median of 1.6 at 48 h. The increase in secretion of IL-1β with MOI was consistent across all *M. tuberculosis* isolates tested in this study. Infections at MOI of 1 and 10 induced similar differential pattern of IL-1beta to MOI 5, although the inter-strain difference in expression was less marked (data not shown).

### 3.2 *M. tuberculosis* isolate-dependent IL-1β induction is not due to altered IL-1β transcription or increased expression of pro-IL-1β

To investigate the possible mechanisms behind the differential IL-1β expression we subdivided the isolates into those which induced low, moderate, or high IL-1β levels, based on quartiles (Figure 2), and selected 3 isolates from each subdivision for further testing.

**Figure 3:**
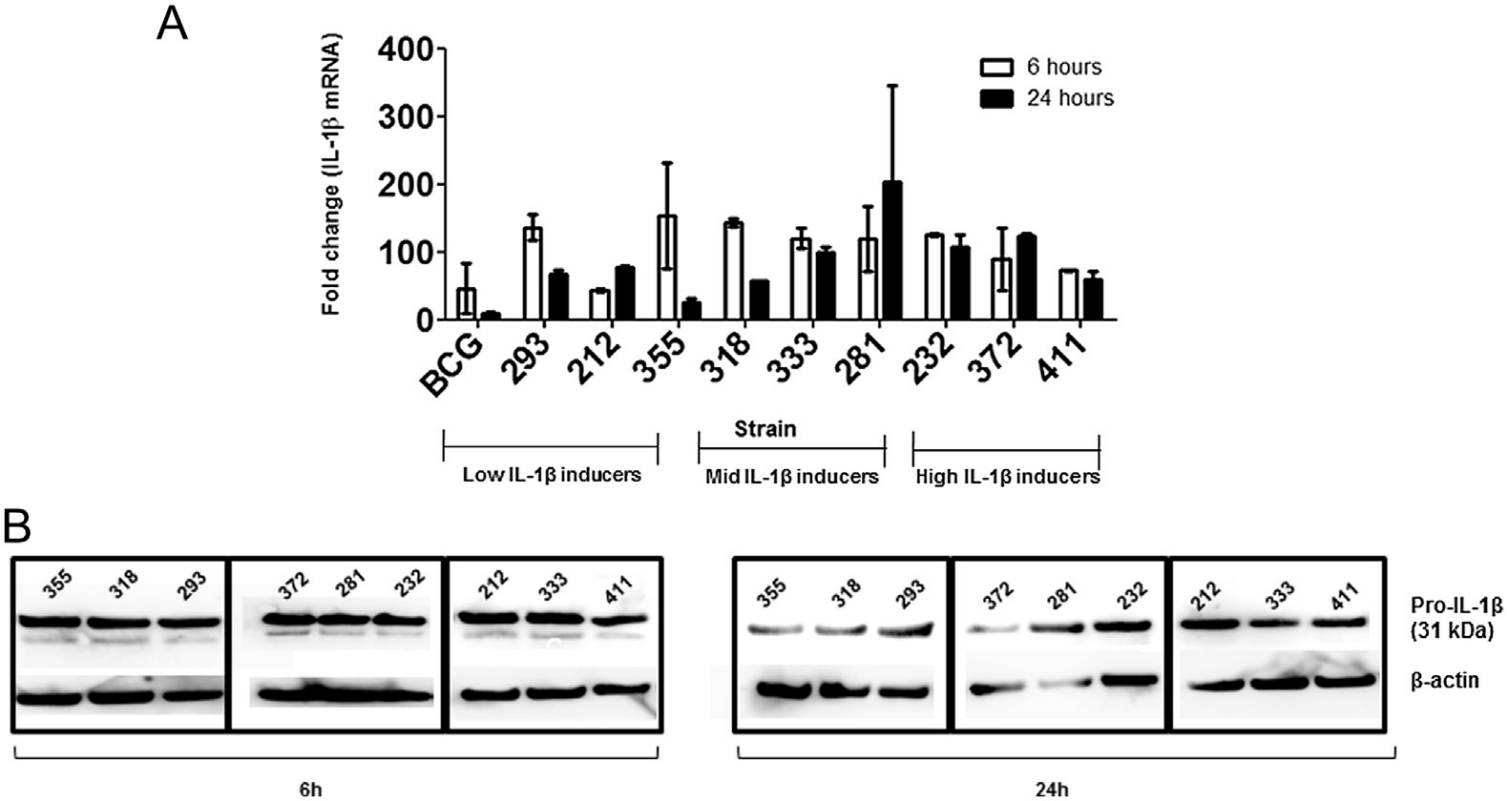
Expression of IL-1β mRNA and pro IL-1β in macrophages is not influenced by the genotype of *M. tuberculosis*. Macrophages were infected with *M. tuberculosis* at a MOI of 5 and (A) expression of IL-1β mRNA at 6 h and 24 h post-infection was determined using real-time PCR. Fold change represents increased IL-1β transcript expression over uninfected cells (B) Levels of pro IL-1β was assayed by western blot at 6 h and 24 h in the lysates of *M. tuberculosis* infected macrophages. For the real-time PCR experiments, data represents means ± SD of two independent experiments.

We first examined whether strains of *M. tuberculosis* induced different concentrations of IL-1β through differential interactions with cell-surface pattern recognition receptors. *M. tuberculosis* infected macrophages rapidly transcribed IL-1β mRNA as early as 2 h post-infection (data not shown) and sustained expression until 24 h (Figure 3A). Additionally, we noted there was no obvious relationship between the fold increase in IL-1β mRNA levels and the amount of IL-1β detected by ELISA. Interestingly, expression of IL-1β mRNA following BCG infection was comparable to some of the clinical *M. tuberculosis* strains. To determine whether post-transcriptional control of IL-1β could account for the observed differences in IL-1β secretion in the supernatants, we profiled the expression of pro-IL-1β in whole cell lysates of macrophages at 6 h and 24 h post-infection by western blot. We found that all strains were capable of activating pro-IL-1β in macrophages (Figure 3B), but pro-IL-1β expression did not differ significantly between strains. These findings suggest that genotype-dependent differences in IL-1β expression are not regulated by differential activation of pattern recognition receptors but via downstream pathways that mediate either the caspase-1-dependent or independent conversion of pro-IL-1β to its active form (Figure 1).

### 3.3 Live bacteria are required to induce optimum IL-1β expression from macrophages

**Figure 4:**
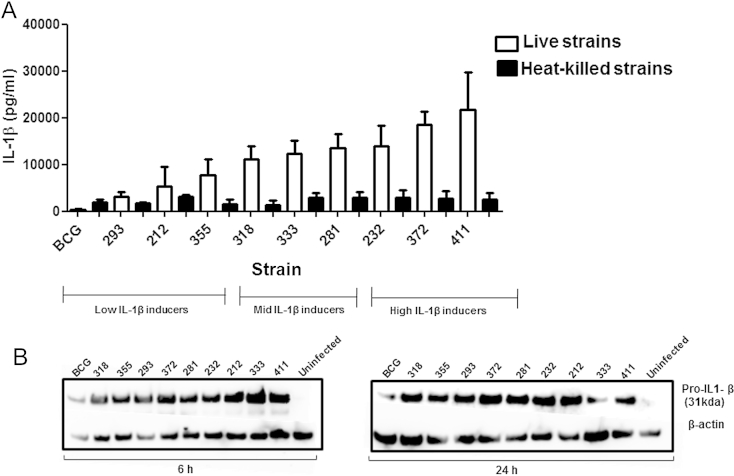
Heat-killed strains of *M. tuberculosis* stimulate less IL-1β secretion from macrophages than viable bacteria. Bone marrow macrophages were stimulated with nine heat-killed strains of *M. tuberculosis* and BCG for 48 h. IL-1β concentrations in the supernatants were determined by ELISA (A). Levels of pro-IL-1β in the cell lysates were assayed by western blot at 6 h and 24 h (B). The cytokine data represents mean ± SD of three independent experiments.

Next, we tested whether IL-1β secretion is dependent on live, viable *M. tuberculosis*. Macrophages were stimulated with heat-killed mycobacteria and the level of IL-1β protein in the supernatant was quantified by ELISA and in the whole cell lysates by western blots. Dead bacteria were less efficient at stimulating secretion of mature IL-1β into the supernatants (Figure 4A) and the differential effect of bacterial genotype on IL-1β expression was lost. In comparison, the viability of the bacteria had no effect on the expression of pro-IL-1β in macrophages (Figure 4B). This suggests that viable *M. tuberculosis* cells actively modulate pro IL-1β maturation pathways to control IL-1β secretion in macrophages.

### 3.4 *M. tuberculosis* driven IL-1β secretion is only partially dependent on caspase-1

**Figure 5:**
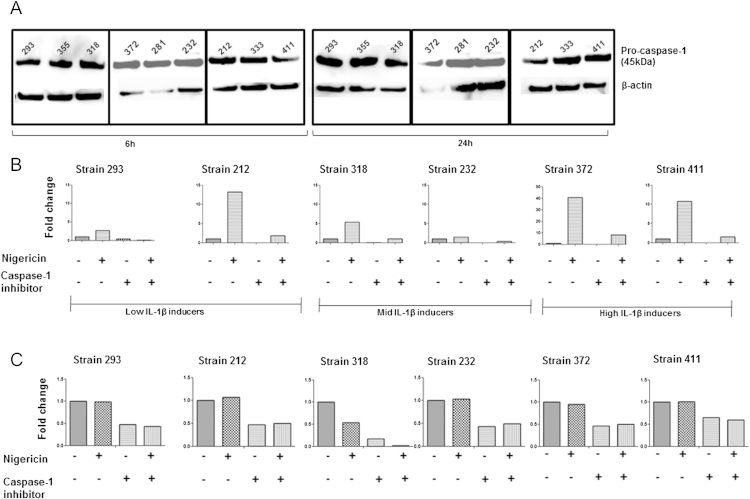
IL-1β secretion is partially dependent on caspase-1. Macrophages were infected with *M. tuberculosis* at a MOI of 5 and (A) levels of pro-caspase-1 were measured by western blot at 6 h and 24 h. Infected macrophages were treated with 20 μM caspase-1 inhibitor (Ac-YVAD-AOM) or treated with 5 μM Nigericin individually or in succession. Cytokine concentrations for IL-1β was analysed in the supernatants by ELISA at 6 h (B) and 48 h (C). The absolute values of IL-1β are expressed as a fold change over control strains.

We hypothesised that differential IL-1β expression may arise due to strain-dependent differences in the activation of caspase-1. First, we compared the expression of pro-caspase-1 between the isolates and found little difference, with the inactive form of caspase-1 being constitutively expressed in macrophages (Figure 5A). We then treated *M. tuberculosis* infected macrophages with caspase-1 inhibitor (YVAD) for a period of up to 48 h, which decreased but did not completely inhibit IL-1β secretion by all the isolates (Figure 5B and C).

Potassium efflux is a common upstream trigger for the activation of the inflammasome, which is required for the activation of caspase-1.^10,26^ Therefore, we assessed if the stimulation of an artificial potassium efflux can rescue IL-1β secretion in macrophages treated with the caspase-1 inhibitor. Macrophages were treated with Nigericin, a known potassium ionophore, and the level of IL-1β was determined in the culture supernatants. There was increased secretion of IL-1β from infected macrophages in response to stimulation with Nigericin at early time points, 6 h (Figure 5B) however, this trend was no longer observed at 48 h (Figure 5C). Following the addition of YVAD, there was approximately a 4-fold reduction in the level of IL-1β release following stimulation of macrophages with Nigericin (Figure 5B). The addition of Nigericin had no effect on the secretion of other pro-inflammatory cytokines such as TNF-α from *M. tuberculosis* infected macrophages (data not shown). These data demonstrate that Nigericin induces the secretion of IL-1β by caspase-1 alone; however, the failure of caspase-1 inhibition to completely inhibit IL-1β secretion suggests *M. tuberculosis* may utilise other caspase-1 independent pathways to induce optimal secretion of IL-1β from macrophages.

### 3.5 Some serine protease inhibitors block *M. tuberculosis* induced secretion of IL-1β in macrophages

**Figure 6:**
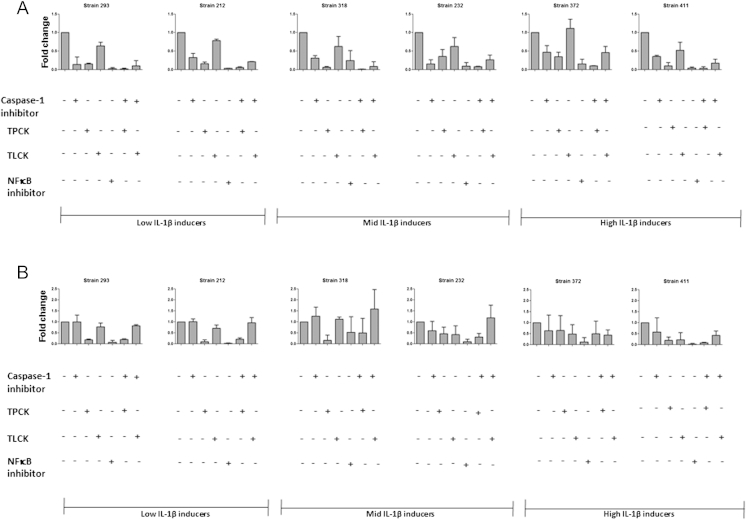
Reduced IL-1β and TNF-α secretion from *M. tuberculosis* infected macrophages in the presence of some serine protease inhibitors. *M. tuberculosis* infected macrophages were treated with 50 μM TPCK, 50 μM TLCK and 20 μM caspase-1 individually or in tandem. Cytokine concentrations for IL-1β (A) and TNF-α (B) were analysed in the supernatants by ELISA at 48 h. The absolute values of cytokines are expressed as a fold change over control strains. Data represents mean ± SD of two independent experiments.

A number of proteases in addition to caspase-1 have been linked to the maturation of IL-1β including serine proteases, which are known to be involved in the alternative-processing pathway leading to the maturation of IL-1β.^27^ We therefore tested inhibitors of serine proteases for their capacity to block IL-1β secretion from *M. tuberculosis* infected macrophages (Figure 6A). These included serine protease inhibitors TPCK, TLCK and AEBSF. Some of these agents can also inhibit NFκB, therefore we also assessed their effect in comparison to formal NFκB inhibition and TNF-α expression (Figure 6B). Treatment of macrophages with TPCK following *M. tuberculosis* infection led to a significant decrease in IL-1β secretion, especially in the low and moderate IL-1β-expressing strains (Figure 6A). This level of inhibition was further enhanced by the addition of TPCK in combination with YVAD, which markedly reduced the secretion of IL-1β with all the *M. tuberculosis* isolates tested (Figure 6A).

**Figure 7:**
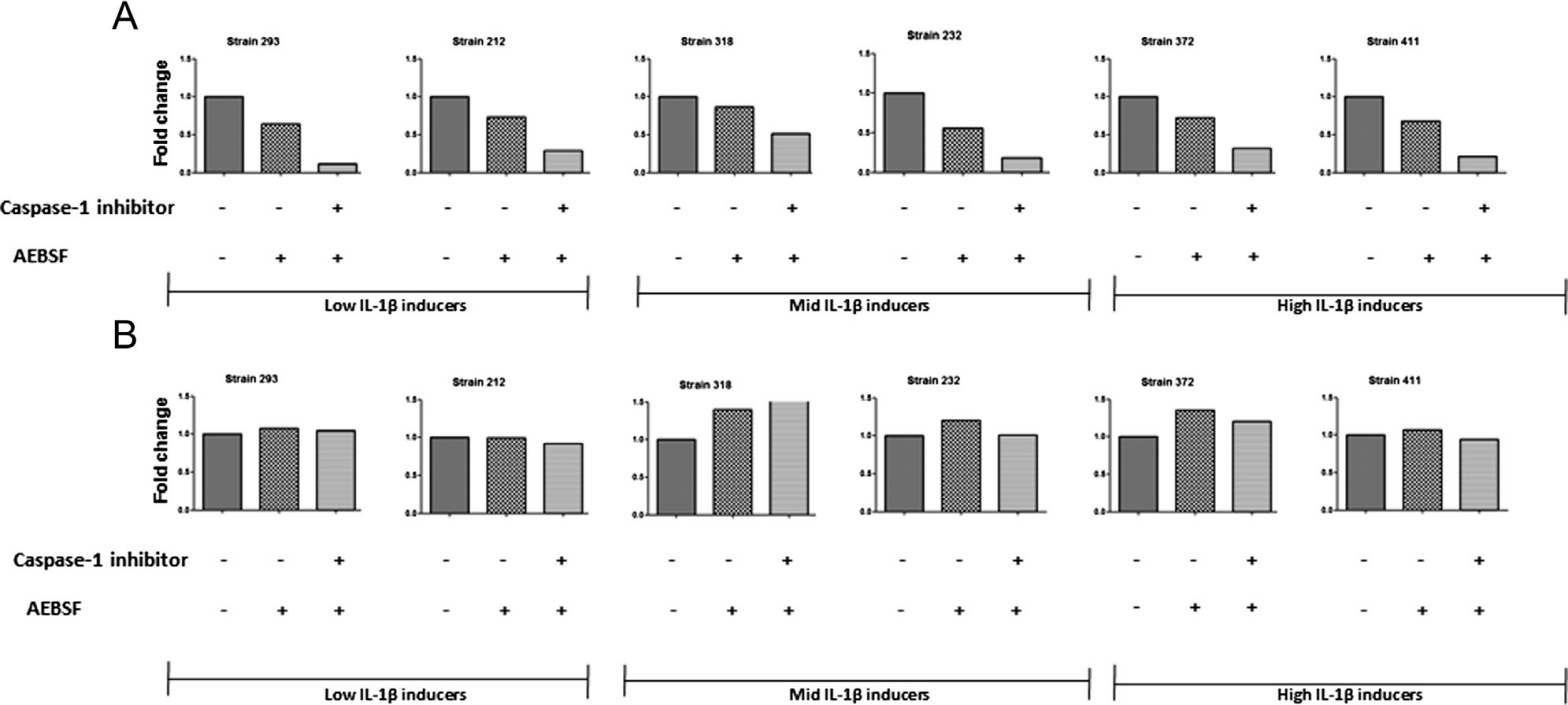
The protease inhibitor AEBSF specifically inhibits IL-1β secretion from *M. tuberculosis* infected macrophages. *M. tuberculosis* infected macrophages were treated with 100 μM AEBSF and 20 μM caspase-1 inhibitor. Cytokine concentrations for IL-1β (A) and TNF-α (B) were analysed in the supernatants by ELISA at 48 h. The absolute values of cytokines are expressed as a fold change over control strains. Data presented is from one experiment only.

The inhibitory effect of TPCK and to a lesser extent TLCK on IL-1β secretion was partially dependent on NF**κ**B inhibition, as they also reduced the release of TNF-**α** from infected macrophages. However, the inhibitor AEBSF specifically inhibited release of only IL-1β (Figure 7A) and not TNF-α (Figure 7B); suggesting AEBSF may specifically target an alternative, protease-dependent pathway of IL-1β expression from *M. tuberculosis*-infected macrophages.

### 3.6 IL-1β production in *M. tuberculosis* infected macrophages does not correlate with host cell death

**Figure 8:**
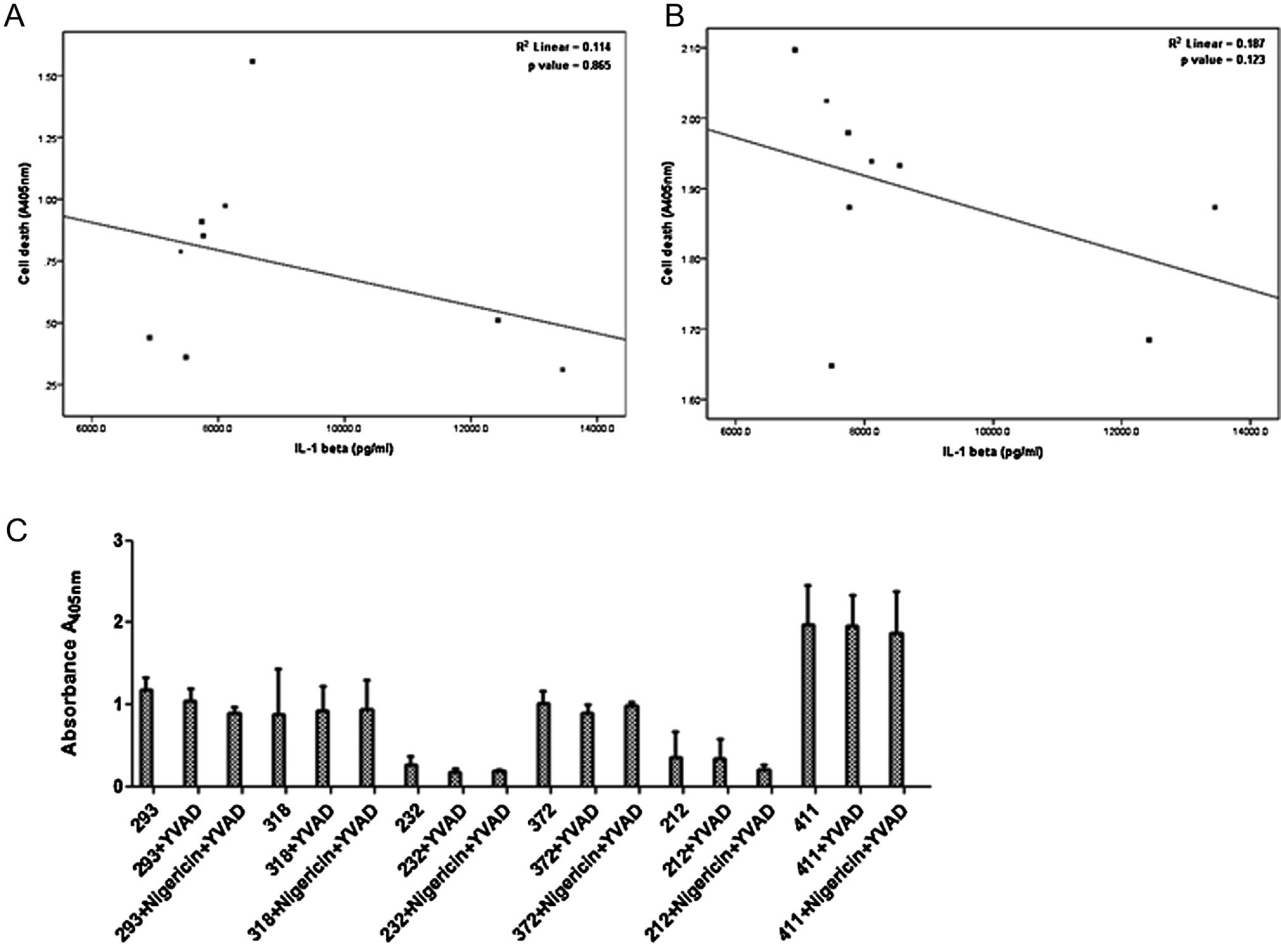
IL-1β secretion from *M. tuberculosis* infected macrophages does not correlate with cell death. Bone marrow derived macrophages were infected with *M. tuberculosis* at a MOI of 5 for 48 h. The amount of necrotic DNA in the cell supernatant (A) and quantity of mono and oligo nucleosomes in the cytoplasmic fraction (B) was determined using ELISA. Infected macrophages were stimulated with 20 μM caspase-1 inhibitor (YVAD) or treated with 5 μM, Nigericin. After 48 h cell death was measured using ELISA (C). Data represents mean ± SD of three independent experiments.

Cell death is an effective innate immune response to *M. tuberculosis* infection. To measure the association between cell death and IL-1β release, we quantified histone associated DNA complexes in the cytoplasmic fraction to determine apoptosis. The supernatant from *M. tuberculosis* infected macrophages was used to quantify necrotic DNA release by ELISA. Strains of *M. tuberculosis* were varied in their ability to cause cell death in macrophages. However, neither the apoptotic (correlation coefficient = −0.552 and *p*-value = 0.123) nor the necrotic cell death (correlation coefficient = 0.067 *p*-value = 0.865) correlated with the amount of IL-1β released from infected macrophages (Figure 8A and B).

To examine if the cell death was dependent on caspase-1, we treated macrophages with a caspase-1 inhibitor and determined cell death in response to *M. tuberculosis* infection. We found that cell death was independent of caspase-1 signalling pathway (Figure 8C). These results suggest that virulence determinants in the strains tested are unlikely to contribute to the differential secretion of IL-1β by manipulating the level of cell death in macrophages.

## 4 Discussion

There is substantially more genetic variation amongst clinical isolates of *M. tuberculosis* than previously considered,^28^ which may contribute to the longstanding appreciation that some strains are more virulent than others, although the mechanisms underpinning this have proved elusive. Macrophages secrete IL-1β in response to *M. tuberculosis* infection,^2,4^ but most of the published work used the laboratory strain H37Rv,^6,25^ which was isolated from a patient with tuberculosis in 1905 and is therefore of questionable relevance to currently circulating clinical isolates of *M. tuberculosis*. Indeed, the variable ability of clinical isolates of *M. tuberculosis* to induce IL-1β secretion from macrophages has not been described.

In this study, we report that clinical isolates of *M. tuberculosis* vary in their capacity to induce IL-1β secretion from macrophages *in vitro*. In agreement with previous reports our data also suggests that IL-1β secretion is dependent on a functional ESX-1 secretion system since BCG failed to induce IL-1β secretion.^6,13,14^ An overview of the panel of screened isolates shows groups of strains that could induce high, moderate or low levels of IL-1β from macrophages. The synthesis of pro-IL-1β and the release of its mature form is a highly regulated multi-step process that includes transcription of the IL-1β gene and its translation into pro-IL-1β, conversion of pro-IL-1β into the mature form, which is finally released into the external environment.^29,30^ It has been reported that the primary pathway of IL-1β induction is regulated at the level of microbial recognition by PRRs involving TLR2/6 and NOD2 signalling pathways.^31^ Our findings reveal that all isolates of *M. tuberculosis* tested are able to up regulate the transcription of the IL-1β mRNA in macrophages, although the levels of the 31 kDa immature form of IL-1β present are similar. Hence it is likely that the difference noted in the secretion of active IL-1β is not regulated by PRRs but by secondary pathways involving the inflammasome and caspase-1 activation.

Secretion of IL-1β is dependent on the viability of the bacteria as heat-killed strains had a substantially reduced capacity to induce secretion. In contrast, it has been reported by others that both heat-killed and viable *M. tuberculosis* H37Rv induced similar levels of IL-1β from infected peripheral blood mononuclear cells.^31^ Conversely, heat-killed *M. marinum* was impaired in its ability to induce IL-1β secretion from bone marrow derived macrophages.^13^ This discrepancy could be explained by differences in experimental setup such as of the mycobacteria used, bacterial burden, and the cell type. Despite these discrepancies, our results strongly support the idea that live *M. tuberculosis* actively modulates signalling pathways downstream of pro-IL-1β formation, to ensure IL-1β secretion into the external environment.

Caspase-1 is a cysteine protease known to play an important role in the cleavage of pro-IL-1β to mature IL-1β.^32^ In our study, inhibition of caspase-1 led to a decrease in IL-1β production by macrophages, consistent with previous studies.^5,25^ However, this inhibition was only partial suggesting a role for an upstream caspase-1 independent pathway for optimal secretion of IL-1β. These findings suggest an incomplete but vital role for caspase-1 in the release of IL-1β *in vitro*. *In vivo*, caspase-1, ASC and NALP3 are shown to be dispensable for the host resistance to *M. tuberculosis* infection.^5,33,34^ Hence, it seems likely that caspase-1 independent pathways play a prominent role in the secretion of IL-1β. An attempt to rescue the partial inhibition of IL-1β by the addition of Nigericin, a known inducer of potassium efflux in macrophages, was unsuccessful. Thus, virulent *M. tuberculosis* activates a potent potassium efflux, which cannot be further enhanced by an external stimulus. Consequently the observed variability of IL-1β secretion from *M. tuberculosis*-infected macrophages cannot be accounted for by the inability of some isolates to induce a potassium efflux.

A marked inhibition of IL-1β release was achieved using the serine protease inhibitors, TPCK, TLCK and AEBSF. Published reports show that the inhibitors TPCK and TLCK are also able to block the activation of NFκB by inhibiting the phosphorylation of IκB.^35,36^ Our results support these observations since the addition of TPCK and to a lesser extent TLCK, led to a decrease in TNF-α secretion, which is also dependent on NFκB activation. Interestingly the protease inhibitor AEBSF had no effect on TNF-α secretion from *M. tuberculosis*-infected macrophages, making it plausible that other serine proteases, independent of caspase-1, play a role in the processing of pro-IL-1β. Further experiments using more targeted approaches such as siRNA will be required to determine the involvement of serine proteases in the processing of pro-IL-1β.

Activation of caspase-1 is dependent on a multi-protein structure, the inflammasome, which is activated by microbial ligands and danger signals. The downstream consequences of inflammasome activation include release of IL-1β/IL-18 and pyroptosis, a form of cell death considered to be dependent on caspase-1 activation.^37^ We demonstrate that IL-1β secretion is accompanied by cell death in macrophages during *M. tuberculosis* infection. Furthermore, the form of cell death observed in our model was unlikely to be pyroptosis, as inhibition of caspase-1 by YVAD had no significant effect on the cell death induced in macrophages; an observation in agreement with previous reports.^25,38^ Based on our observation of the enrichment of oligonucleosomes in the cytoplasmic fraction of cell lysates, we suggest that it is apoptotic cell death. Macrophage apoptosis represents a key component of the host immune response to *M. tuberculosis* infection, which leads to a decrease in mycobacterial viability, a mechanism favoured by the host to remove and clear the pathogen. However, we found infection of macrophages with *M. tuberculosis* also led to increased levels of necrotic DNA in the supernatant. Previous studies have reported that bacterial burden is a crucial factor that determines the type of cell death induced in *M. tuberculosis* infected macrophages^25,39^; it is likely that key factors such as the genetic background of the infecting bacterial strain also influence the type of cell death.^40,41^ Overall, the findings suggest that cell death accompanies the release of IL-1β but it is unlikely to be the determining factor for the differential induction of IL-1β observed in *M. tuberculosis* infected macrophages.

ESX-1 substrates like ESAT-6 are reported to be involved in the activation of caspase-1.^16^ From sequencing analysis, we could not detect any sequence variation in *esxA* (Rv3875) or *esxB* (Rv3874), the genes encoding ESAT-6 and CFP-10 (unpublished data). This observation agrees with two recent studies that assessed the genetic diversity in a large collection of clinical isolates of *M. tuberculosis*. These studies reported that the lack genetic variation in *esxA* and *esxB* likely confirms the conserved nature of these proteins.^42,43^ Consequently, it is unlikely that strain-dependent differences in IL-1β secretion observed here are due to changes in EsxA or EsxB.

Enzymes other than caspase-1 have been reported to cleave pro-IL-1β into the mature form. Neutrophil derived serine proteases, aspartyl proteases from *Candida albicans* and cysteine proteases from *Streptococcus pyogenes* all directly cleave IL-1β.^44,45^ Thus, it is plausible that mycobacterial proteases might also be capable of processing pro-IL-1β, and future studies will address this possibility.

In conclusion, we demonstrate that macrophages infected with a panel of clinical isolates of *M. tuberculosis* secrete IL-1β. The pathways that control secretion of IL-1β are dependent on both caspase-1 and serine proteases. Although cell death is an important consequence of *M. tuberculosis* infection of macrophages, it does not seem to play a role in the differential secretion of IL-1β. Further delineation of the protease-dependent pathways will provide an insight into the complex regulatory mechanisms that control IL-1β secretion in *M. tuberculosis* infected macrophages.
